# Analysis and Simulation Verification of the Verticality Measurement Model for Single Offshore Pile Based on Binocular Vision

**DOI:** 10.3390/s25237374

**Published:** 2025-12-04

**Authors:** Shaohui Li, Yanlong Zhu, Yuanyuan Cao, Xinghua Li, Zhenjie Zhou

**Affiliations:** 1 Tianjin Research Institute for Water Transport Engineering, M.O.T., Tianjin 300456, China; lishh@tiwte.ac.cn (S.L.); yanlong_zhu_2009@163.com (Y.Z.); caoyy@tiwte.ac.cn (Y.C.); 2 School of Precision Instrument and Opto-Electronics Engineering, Tianjin University, 92 Weijin Road, Tianjin 300072, China; lixinghua@tju.edu.cn

**Keywords:** offshore wind power, single pile, binocular vision, ship posture, verticality

## Abstract

Accurately measuring the verticality of a single pile is of crucial importance for ensuring the safe operation of offshore wind power projects. However, mainstream methods have disadvantages such as high dependence on manual labor, low real-time performance, and susceptibility to construction site conditions and marine environmental impacts. The method of measuring the verticality of a single offshore pile based on binocular vision is one of the emerging measurement methods, but there is currently a lack of research on measurement models. In order to clarify the principle of the method for measuring the verticality of a single pile at sea based on binocular vision, this paper starts from the imaging principle of the camera and studies and derives the measurement model of the verticality of a single pile in the global coordinate system and the error model of the measurement system. To verify the correctness of the model and method, a testing experimental platform was built to simulate the measurement of the ship under static and dynamic conditions, and the measurement results were compared with those of the total station. The experimental results show that in the static simulation experiment, the maximum absolute error of the verticality of a single pile is 0.2°, the maximum absolute error of the roll angle is 0.3°, and the maximum absolute error of the pitch angle is 0.3°. In the dynamic simulation experiment, the maximum absolute error of the verticality of a single pile is 0.4°, the maximum absolute error of the roll angle is 0.3°, and the maximum absolute error of the pitch angle is 0.3°. This paper verified the correctness of the model and provided model support for measuring the verticality of single piles at sea.

## 1. Introduction

In the current energy crisis and increasingly serious environmental problems, wind energy, as a pollution-free and clean energy source, has enormous potential for development. Offshore wind power, as an important component of clean energy, plays a crucial role in the global energy structure transformation [1]. Single pile is the most commonly used supporting structure for offshore wind power, and its verticality relative to the standard horizontal plane directly affects the stability and long-term reliability of wind turbines [2]. Therefore, accurately measuring the verticality of a single pile is of crucial importance for ensuring the safe operation of offshore wind power projects.

The verticality measurement technology for large single-pile structures can be primarily divided into four methods based on the different measuring instruments used: the inclinometer measurement method, total station measurement method, 3D laser scanning method, and visual measurement method.

The inclinometer measurement method involves installing an inclinometer on a single-pile structure and using data transmission technology to return and analyze the measured data to monitor the verticality information of the target pile. Wu H [3] analyzed the current situation of wind turbine fault detection at home and abroad, and designed a wind turbine tower tilt monitoring device based on STM32 microcontroller, ADXL345 tilt sensor, and LoRa/WiFi technology. It is characterized by strong anti-interference ability, long transmission distance, and low power consumption. Koo S [4] proposed an automatic verticality securing system for large circular steel pipes based on a verticality monitoring system. The experimental results prove that the system runs well and secures the verticality of less than 0.2° in the suction bucket-based model experiment.

The total station measurement method involves emitting a single laser beam towards a single target pile and receiving the light signal reflected back from the surface of the object. After analysis by the high-precision processing system inside the total station, the angle and distance information of the target point are obtained. Data from multiple target points is comprehensively analyzed to further obtain the verticality of a single pile. Liao J [5] uses a two-point measurement station method based on a prism free total station to quickly assess the tilting of problem wind turbines caused by concrete damage. Mladen Zrinjski et al. [6] used a total station sampling method to fit the cross-sectional ellipses of chimneys of different heights, and then used polynomial fitting to derive the axis information of the chimney to determine the verticality of the entire chimney structure.

The 3D laser scanning method collects and records high-density point cloud data by emitting a dense laser beam towards a single target pile, capturing the reflected light from the pile surface. Marjetič [7] and Barazzetti [8] captured the point cloud coordinates of the chimney using 3D laser scanning technology, processed the point cloud data on the same horizontal plane using the least squares method, fitted the 3D vector of the chimney’s central axis, and calculated the verticality of the chimney.

The visual measurement method refers to the use of a camera to capture surface features of a single pile, and the use of image processing techniques to analyze the position, shape, and other characteristic information of the single pile. Combined with camera parameter data, it achieves the measurement of the verticality of the single pile. Xue Wanting [9] designed a verticality measurement system for land wind turbine towers, which uses a line laser to project laser lines as reference lines on the wind turbine tower. The system captures tower images with a camera and processes them. Combined with a laser rangefinder to obtain the relative distance between the camera and the tower, the system calculates the size and position of the cross-sectional circle at different heights of the wind turbine tower, completes the three-dimensional reconstruction of the wind turbine tower, and measures the verticality of the tower. Fugro, a foreign company [10], has conducted earlier research in the field of monitoring the verticality of single piles at sea. The company has independently developed a high-precision and highly intelligent binocular vision monitoring system, which is specifically used to detect the verticality of single piles at sea. In practical applications, Fugro has accumulated a wealth of experience in monitoring the verticality of single piles.

The inclinometer measurement method can achieve remote monitoring of the verticality information of single piles at sea, with low installation cost and high accuracy of the obtained data. However, due to the sinking of a single pile at sea and the significant vibration generated by the pile body during the pile driving process, the installation of the inclinometer needs to consider structural stability and ensure that the inclinometer is aligned horizontally with the flange, which increases the difficulty of installation. The total station measurement method requires a high installation position for the instrument, which needs to be placed in a relatively stationary position relative to the standard horizontal plane, usually on the pile positioning platform or pile driving hull. However, the positioning platform space is usually narrow, and the pile driving vessel generates strong vibrations during engineering operations, requiring operators to adjust equipment parameters in real time and continuously operate the total station to obtain information from multiple measurement points, thereby increasing workload and reducing measurement efficiency. Although 3D laser scanning technology provides high-precision and fast measurement results, its expensive equipment and complex data processing process limit its widespread use.

Binocular stereo vision technology is widely used due to its high measurement accuracy, strong real-time performance, low cost, high efficiency, and non-contact measurement advantages [11]. As an emerging application technology, it is crucial to study and analyze its measurement model. In order to clarify the principle of a binocular vision-based method for measuring the verticality of a single pile at sea, this paper starts from the imaging principle of a camera, studies the three-dimensional vector measurement model of a single pile in the ship’s coordinate system and the mapping model from the ship’s coordinate system to the global coordinate system, and derives a measurement model for the verticality of a single pile in the global coordinate system. To verify the correctness of the model, a testing experimental platform was built to simulate the verticality measurement experiment of a single pile on a ship under static and dynamic conditions. Compare the experimental results with the measurement results of the total station to verify the correctness of the measurement model.

## 2. Measurement Principle

The verticality of a single pile refers to the degree of proximity between the angle of the single pile relative to the standard horizontal plane and the ideal 90-degree angle. In practical engineering projects, to ensure that a single pile is in a standard vertical state, a global coordinate system is usually established on the standard horizontal plane. The roll angle and pitch angle are used to measure the degree of inclination of the single pile relative to a specific direction, providing reliable data reference for adjusting the verticality of the single pile to ensure that it meets the requirements of engineering specifications.

**Figure 1:**
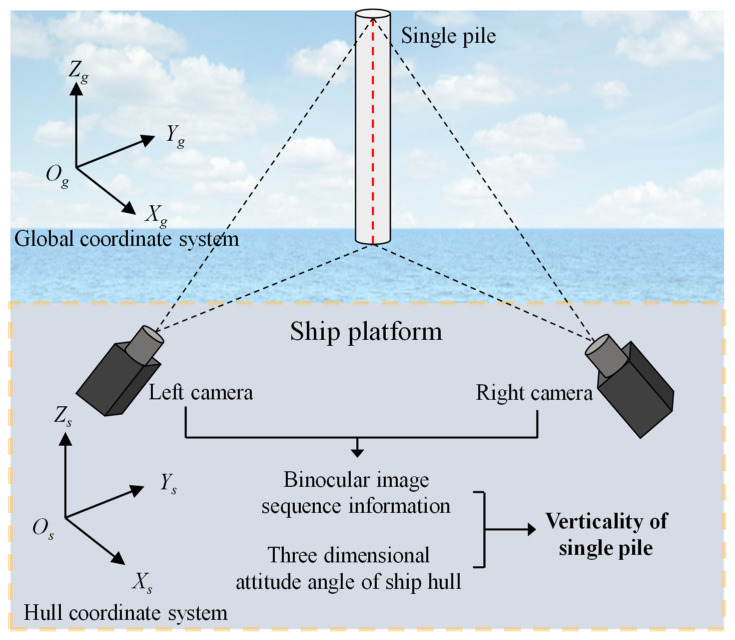
Layout of measurement system structure.

The overall structural layout of the single pile verticality measurement system based on binocular vision is shown in Figure 1, including 2 industrial cameras, 1 industrial computer, ship 3D attitude angle output equipment, and corresponding equipment support structures.

**Figure 2:**
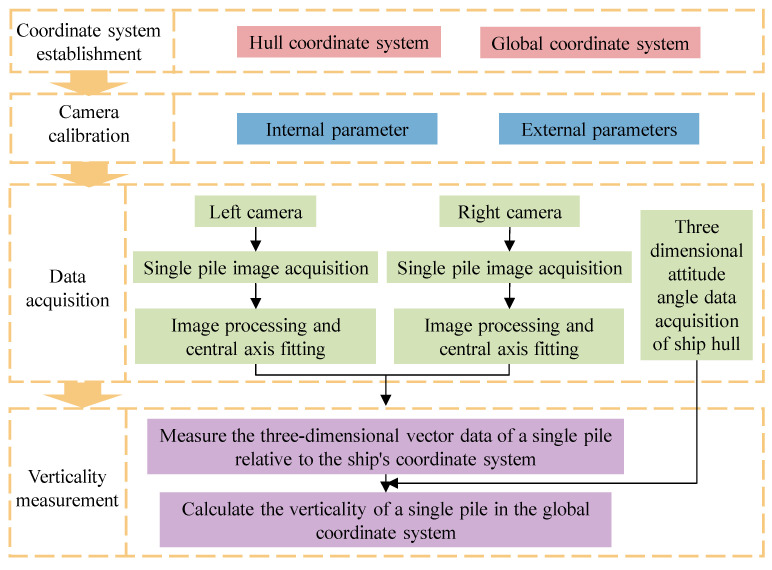
Design of single-pile verticality measurement process.

The measurement process includes four stages: coordinate system establishment, camera calibration, data acquisition, and verticality measurement. The measurement process is shown in Figure 2.

## 3. Measurement Model Analysis

A deep understanding of the imaging model of monocular cameras is the foundation for exploring the principles of binocular vision imaging. By analyzing the binocular vision imaging model in the hull coordinate system, the three-dimensional vector of a single pile can be determined. Establishing a mapping model from the hull coordinate system to the global coordinate system, combined with the three-dimensional attitude angle of the ship itself, can map the three-dimensional vector information of a single pile in the hull coordinate system to the global coordinate system, thereby obtaining the verticality data of the single pile.

### 3.1. Camera Imaging Model

**Figure 3:**
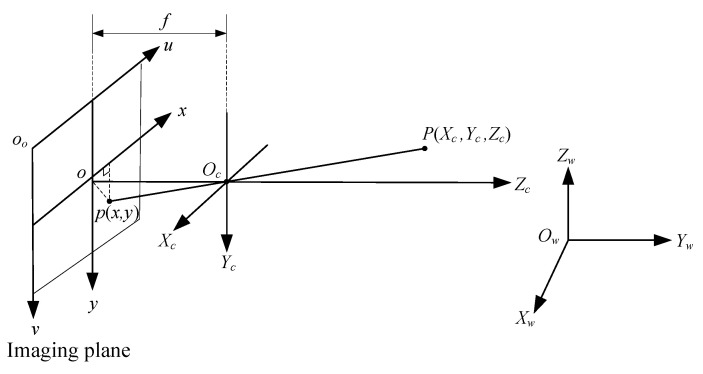
Camera pinhole imaging model.

In the process of camera imaging, the three-dimensional points in space are linearly related to the projection points in the camera imaging plane, and satisfy the principle of pinhole imaging. The coordinate transformation from three-dimensional spatial points to image imaging points involves four coordinate systems [12], as shown in Figure 3.

The world coordinate system $O_{w}\text{-}X_{w}Y_{w}Z_{w}$ provides the absolute positions of spatial points in the real world, and the coordinate axes follow the right-hand rule. The camera coordinate system $O_{c}\text{-}X_{c}Y_{c}Z_{c}$ describes the position of a spatial point relative to the camera, measured in millimeters. It is a three-dimensional coordinate system established with the camera’s optical center *O_c_* as the reference point and *O_c_Z_c_* as the main optical axis of the camera. The image coordinate system *o*-*xy* is used to describe points on the camera image plane, in millimeters. *o* is the principal point where the camera optical axis intersects the imaging plane. The *ox* and *oy* axes of the image coordinate system are parallel to the *O_c_X_c_* and *O_c_Y_c_* axes of the camera coordinate system, respectively. The pixel coordinate system *o_o_*-*uv* is used to locate pixels in digital images, with the unit being pixel. The coordinate origin of the pixel coordinate system is located in the upper left corner, with *o_0_*-*u* parallel to *ox* horizontally to the right and *o_0_*-*v* parallel to *oy* vertically downward.

The spatial point *P* is mapped to the point *p* on the actual imaging plane through pinhole imaging. In the world coordinate system, the coordinates of the spatial point *P* are (X*_w_*, Y*_w_*, Z*_w_*). In the pixel coordinate system, the coordinates of the imaging point *p* are (*u*, *v*). According to the conversion relationship between different coordinate systems during camera imaging, the formula for the world coordinates of spatial point *P* to the pixel coordinates of imaging point *p* are as follows [13]:

(1) $$Z_{c}\left[\begin{array}{c} u\\ v\\ 1 \end{array}\right]=\left[\begin{array}{cccc} \frac{f}{dx} & 0 & u_{0} & 0\\ 0 & \frac{f}{dy} & v_{0} & 0\\ 0 & 0 & 1 & 0 \end{array}\right]\left[\begin{array}{cc} R & T\\ 0^{T} & 1 \end{array}\right]\left[\begin{array}{c} X_{w}\\ Y_{w}\\ Z_{w}\\ 1 \end{array}\right]=M_{1}M_{2}\left[\begin{array}{c} X_{w}\\ Y_{w}\\ Z_{w}\\ 1 \end{array}\right]$$

In the formula, *Z_c_* represents the component of spatial point *P* on the *O_c_Z_c_* axis in the camera coordinate system. *f* represents the focal length from the camera’s optical center to the imaging plane, measured in millimeters. *dx* and *dy*, respectively, represent the physical dimensions of a unit pixel in the x-axis and y-axis directions of the image, measured in millimeters per pixel. (*u*_0_, *v*_0_) represents the principal point coordinates in the image pixel coordinate system; *R* and *T*, respectively, represent the rotation matrix and translation matrix from the world coordinate system to the camera coordinate system, *M*_1_ represents the internal parameter matrix of the camera, and *M*_2_ represents the external parameter matrix of the camera. *M*_1_*M*_2_ represents the imaging matrix of the camera.

### 3.2. Three-Dimensional Vector Measurement Model of Single Pile in Hull Coordinate System

Based on binocular vision imaging technology, the three-dimensional vector of a single pile relative to the ship’s coordinate system is measured. The specific solving process is divided into two steps: first, the normal vector of the object image plane along the axis of the single pile in the ship’s coordinate system is solved. Then, the two normal vectors solved are cross-multiplied to obtain the three-dimensional vector of the single pile in the ship’s coordinate system.

#### 3.2.1. Solving the Normal Vector of the Object Image Plane in a Monocular Camera

**Figure 4:**
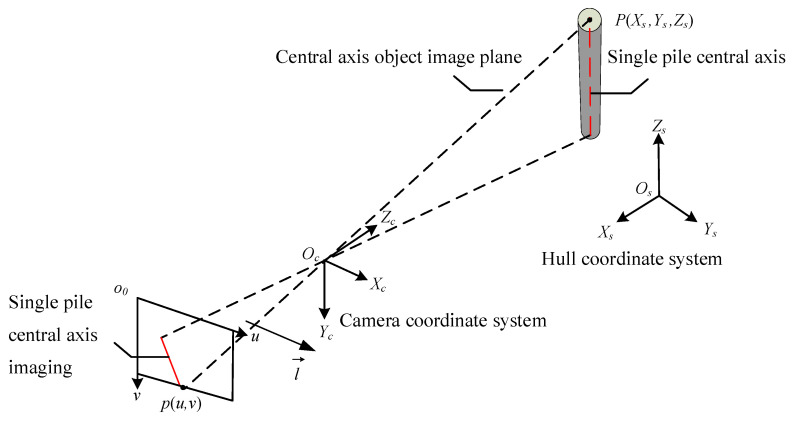
Schematic diagram of monocular camera imaging.

The object image plane of the central axis refers to the plane formed by the central axis of a single pile and the image. As shown in Figure 4, using the hull coordinate system as the world coordinate system, assuming the hull coordinate system is *O_s_*-*X_s_Y_s_Z_s_*, there is a point *P* (*X_s_*, *Y_s_*, *Z_s_*) on the axis of the single pile, corresponding to point *p* (*u*, *v*) in the camera pixel coordinate system *o*_0_-*uv*.

According to Equation (1), the mapping relationship between the spatial point *P* in the hull coordinate system and the imaging point *p* in the pixel coordinate system is

(2) $$Z_{c}\left[\begin{array}{c} u\\ v\\ 1 \end{array}\right]=\left[\begin{array}{cccc} m_{11} & m_{12} & m_{13} & m_{14}\\ m_{21} & m_{22} & m_{23} & m_{24}\\ m_{31} & m_{32} & m_{33} & m_{34} \end{array}\right]\left[\begin{array}{c} X_{s}\\ Y_{s}\\ Z_{s}\\ 1 \end{array}\right]$$

In the formula, *m*_11_~*m*_34_ are the elements of the camera imaging matrix. Assuming that the straight line expression of the central axis of a single pile in the camera pixel coordinate system is *u* = *kv* + *b*, we can obtain

(3) $$\frac{m_{11}X_{s}+m_{12}Y_{s}+m_{13}Z_{s}+m_{14}}{m_{31}X_{s}+m_{32}Y_{s}+m_{33}Z_{s}+m_{34}}=k\frac{m_{21}X_{s}+m_{22}Y_{s}+m_{23}Z_{s}+m_{24}}{m_{31}X_{s}+m_{32}Y_{s}+m_{33}Z_{s}+m_{34}}+b$$

The normal vector of the central axis image plane is

(4) $$\vec{l}=\left(km_{21}+bm_{31}-m_{11},km_{22}+bm_{32}-m_{12},km_{23}+bm_{33}-m_{13}\right)$$

According to Equations (2) and (4), it can be inferred that the normal vector of the central axis object image plane is independent of the translation matrix from the hull coordinate system to the camera coordinate system.

#### 3.2.2. Obtaining the Three-Dimensional Vector of a Single Pile in the Hull Coordinate System

**Figure 5:**
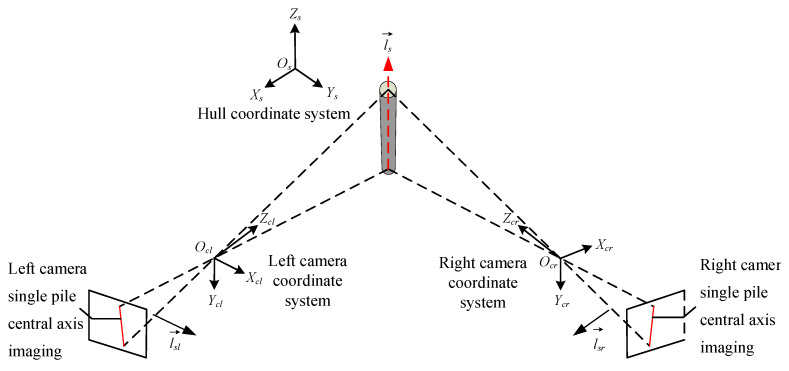
Schematic diagram of binocular camera single-pole imaging.

When the world coordinate system of the left and right cameras is the hull coordinate system, the three-dimensional vector of the single pile’s central axis in the hull coordinate system can be obtained by cross-multiplying the normal vectors of the central axis object plane from the perspectives of the two cameras, as shown in Figure 5.

The normal vectors of the plane formed by the imaging of the single-pile axis and the left and right camera pixels in the hull coordinate system are $\vec{l_{sl}}$ and $\vec{l_{sr}}$, respectively. Therefore, the expression of the three-dimensional vector $\vec{l_{s}}$ of the single-pile axis in the hull coordinate system is

(5) $$\vec{l_{s}}=\vec{l_{sl}}\times \vec{l_{sr}}=\left(l_{sx},l_{sy},l_{sz}\right)$$

In the formula, $l_{sx}$, $l_{sy}$, and $l_{sz}$, respectively, represent the components of the three-dimensional vector $\vec{l_{s}}$ on the *O_s_X_s_*, *O_s_Y_s_*, and *O_s_Z_s_* axes of the hull coordinate system.

In summary, according to the principle of binocular vision imaging, it is possible to obtain the normal vectors of the single-pile axis object plane under binocular cameras separately. By cross-multiplying these two normal vectors, the three-dimensional vector of the single pile in ship coordinates can be obtained. Since the vector is a vector that only contains direction and magnitude information, it is independent of the translation matrix between different coordinate systems [14]. Therefore, the three-dimensional coordinate system established in this article and the conversion between coordinate systems only consider the rotational relationship, while the translational relationship can be ignored.

### 3.3. Mapping Model from Hull Coordinate System to Global Coordinate System

Establish the hull coordinate system $O_{s}\text{-}X_{s}Y_{s}Z_{s}$ and at the same time establish the reference coordinate system $O_{s\prime }\text{-}X_{s\prime }Y_{s\prime }Z_{s\prime }$ with the $O_{s\prime }X_{s\prime }Y_{s\prime }$ plane parallel to the standard horizontal plane. The angle between the $O_{s}X_{s}$ axis and the standard horizontal plane is denoted as the roll angle *α*, and the angle between the $O_{s}Y_{s}$ axis and the standard horizontal plane is denoted as the pitch angle *β*. The coordinate origin $O_{s}$ of the hull coordinate system coincides with the coordinate origin $O_{s\prime }$ of the reference coordinate system, and the projection of the $O_{s}X_{s}$ axis on the horizontal plane coincides with the $O_{s\prime }X_{s\prime }$ axis.

Let the hull coordinate system rotate w around $O_{s}X_{s}$, and then rotate ϕ around $O_{s}Y_{s}$ to obtain the reference coordinate system. The global coordinate system is set to $O_{g}\text{-}X_{g}Y_{g}Z_{g}$. The $O_{g}X_{g}Y_{g}$ plane is parallel to the standard horizontal plane, and the $O_{g}Z_{g}$ axis is opposite to the direction of gravity. The angle between the $O_{s\prime }X_{s\prime }$ axis of the reference coordinate system and the $O_{g}Z_{g}$ axis is the yaw angle κ.

The rotation matrix from the hull coordinate system to the global coordinate system is [15,16]

(6) $$R_{sg}=\left[\begin{array}{ccc} \cos\kappa \cos\varphi & \cos\kappa \sin\varphi \sin\omega -\sin\kappa \cos\omega & \sin\kappa \sin\omega +\cos\kappa \sin\varphi \cos\omega \\ \sin\kappa \cos\varphi & \cos\kappa \cos\omega +\sin\kappa \sin\varphi \sin\omega & \sin\kappa \sin\varphi \cos\omega -\cos\kappa \sin\omega \\ -\sin\varphi & \cos\varphi \sin\omega & \cos\varphi \cos\omega \end{array}\right]$$

### 3.4. Vertical Measurement Model of Single Pile in Global Coordinate System

**Figure 6:**
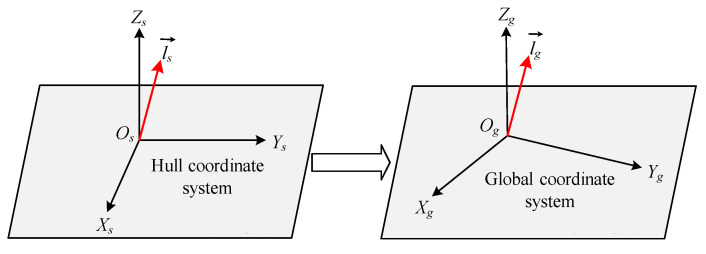
The conversion model from hull coordinate system to global coordinate system.

The three-dimensional vector $\vec{l_{s}}$ of a single pile in the hull coordinate system was obtained in Section 3.2, and the rotation matrix $R_{sg}$ from the hull coordinate system to the global coordinate system was obtained in Section 3.3. The transformation model of the three-dimensional vector of a single pile in the hull coordinate system to the global coordinate system is shown in Figure 6.

According to the rotation transformation between coordinate systems, the three-dimensional vector $\vec{l_{g}}$ of a single pile in the global coordinate system is

(7) $$\vec{l_{g}}=R_{sg}\vec{l_{s}}$$

**Figure 7:**
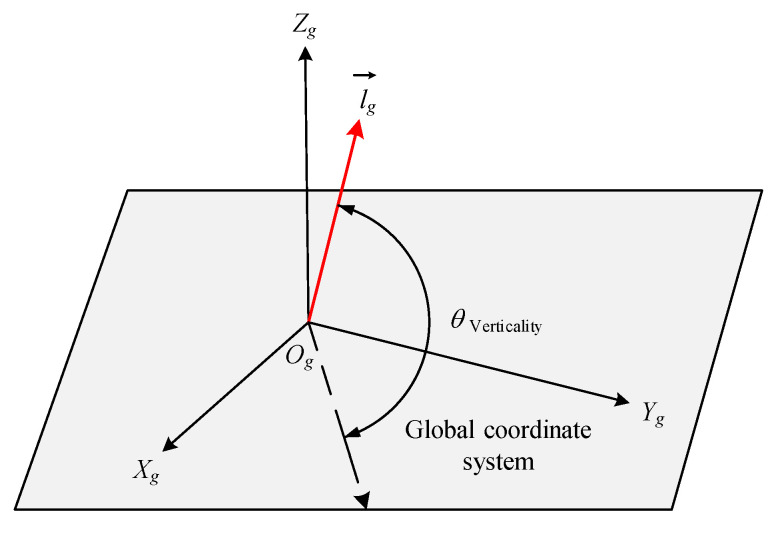
Schematic diagram of verticality in global coordinate system.

In the formula $\vec{l_{g}}=\left(l_{gx},l_{gy},l_{gz}\right)$, $l_{gx},l_{gy},l_{gz}$ respectively represent the components of the three-dimensional vector $\vec{l_{g}}$ on the $O_{g}X_{g}$, $O_{g}Y_{g}$, and $O_{g}Z_{g}$ axes of the global coordinate system. Therefore, the verticality q of a single pile is shown in Equation (8), and the schematic diagram of the verticality of a single pile is shown in Figure 7.

(8) $$\theta_{\mathrm{Verticality}}=\arctan\left(\frac{\left|l_{gz}\right|}{\sqrt{l_{gx}^{2}+l_{gy}^{2}}}\right)$$

**Figure 8:**
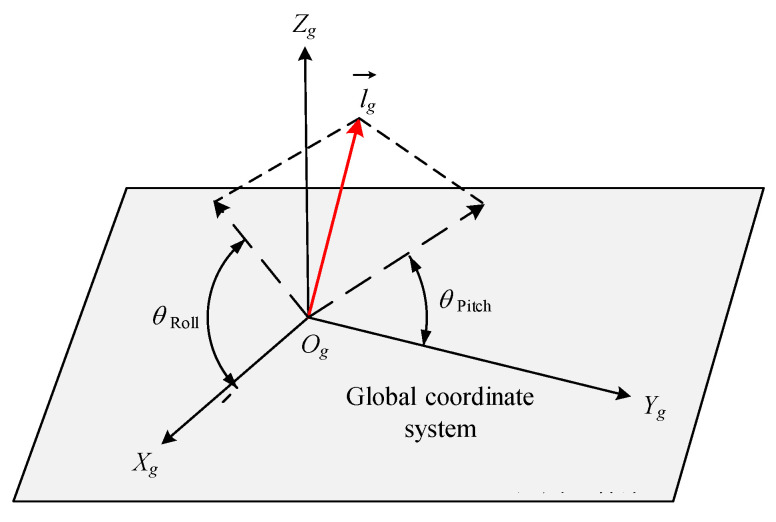
Schematic diagram of single-pile roll angle and pitch angle in global coordinate system.

In the actual engineering operation process, in order to adjust the verticality of a single pile to meet the engineering standards, it is necessary to decompose its verticality in the global coordinate system into roll angle and pitch angle, in order to obtain the tilt angle of the single pile in a specific direction and adjust its verticality to reach the predetermined angle. The roll angle $\theta_{\mathrm{Roll}}$ and pitch angle $\theta_{\mathrm{Pitch}}$ of a single pile are shown in Equation (9), and the schematic diagram of the roll angle and pitch angle of a single pile is shown in Figure 8.

(9) $$\left\{\begin{array}{c} \theta_{\mathrm{Roll}}=\arctan\left(\frac{l_{gz}}{l_{gx}}\right)\\ \theta_{\mathrm{Pitch}}=\arctan\left(\frac{l_{gz}}{l_{gy}}\right) \end{array}\right.$$

In the process of calculating the three-dimensional vector $\vec{l_{g}}$, there is no constraint on its direction, which may result in two vector solutions with opposite directions. But there is no difference in the results for verticality $\theta_{\mathrm{Verticality}}$, roll angle $\theta_{\mathrm{Roll}}$, and pitch angle $\theta_{\mathrm{Pitch}}$ of the single pile calculated using these two solutions, so there is no need to consider the influence of different solutions.

### 3.5. Error Model of Measurement System

**Figure 9:**
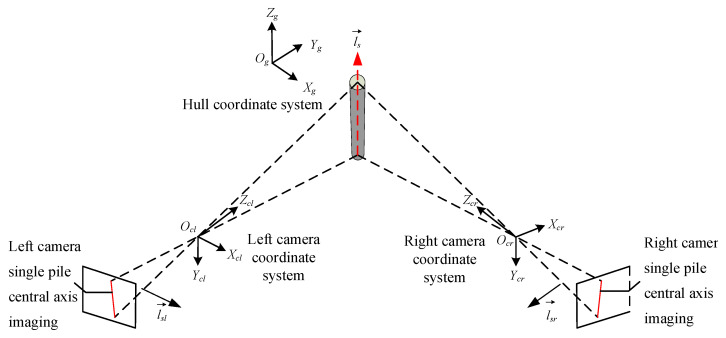
Binocular camera imaging model.

In order to investigate the effect of the optical axis angle between binocular cameras on the verticality measurement of a single pile, assuming that the hull coordinate system is consistent with the global coordinate system, an ideal binocular camera model is established for analysis, as shown in Figure 9. Assuming there is an error in the single axis obtained from the image of the left camera, and no error in the single axis obtained from the image of the right camera, the optical axis of the left camera intersects with the central axis of the single pile, and the optical center of the right camera forms a plane parallel to the $X_{g}O_{g}Z_{g}$ plane with the central axis of the single pile [17].

The central axis angle error and displacement error obtained from the image of the left camera are set as Δθ and Δr, respectively, and the central axis equation is expressed as

(10) $$x\cos\left(\theta +\Delta \theta \right)+y\sin\left(\theta +\Delta \theta \right)=r+\Delta r$$

Let θ and *r* both be 0. Let the angle between the left camera’s optical axis and the $X_{g}O_{g}Y_{g}$ plane be *α*, and the angle between the projection of the optical axis on the $X_{g}O_{g}Y_{g}$ plane and the $O_{g}Y_{g}$ axis be *β*. The intersection of the left and right camera’s optical centers with the plane formed by the single pile’s central axis in the global coordinate system yields the angle error *w* between the single pile’s central axis vector and the ideal central axis vector.

(11) $$\tan\omega =\frac{\sin\left(\Delta \theta \right)\cos\left(a\right)+\frac{\Delta r}{f}\sin\left(a\right)}{\cos\left(\Delta \theta \right)\cos\left(\beta \right)+\sin\left(\Delta \theta \right)\cos\left(a\right)\sin\left(\beta \right)-\frac{\Delta r}{f}\cos\left(a\right)\sin\left(\beta \right)}$$

In the formula, ω refers to the verticality error.

The impact on perpendicularity error is discussed separately.

#### 3.5.1. The Impact of Δθ on Verticality Error

Let Δr=0, and Equation (11) can be expressed as

(12) $$\tan\omega =\frac{\sin\left(\Delta \theta \right)\cos\left(a\right)}{\cos\left(\Delta \theta \right)\cos\left(\beta \right)+\sin\left(\Delta \theta \right)\cos\left(a\right)\sin\left(\beta \right)}$$

Due to the small value of Δθ, $\cos\left(\Delta \theta \right)\gg \sin\left(\Delta \theta \right)$, then

(13) $$\tan\omega \approx \tan\left(\Delta \theta \right)\frac{\cos\left(a\right)}{\cos\left(\beta \right)}$$

When *β* approaches 90°, the error generated by Δθ will be very large. When *β* = 0°, that is, the two camera optical axes intersect orthogonally, and *w* and Δθ are close to the same order of magnitude, it can be seen that the closer the angle between the two camera optical axes is to 90°, the smaller the measurement error.

#### 3.5.2. The Impact of Δr on Verticality Error

Let Δθ=0°, and Equation (11) can be expressed as

(14) $$\tan\omega =\frac{\frac{\Delta r}{f}\sin\left(a\right)}{\cos\left(\beta \right)-\frac{\Delta r}{f}\cos\left(a\right)\sin\left(\beta \right)}$$

Due to Δr/f≪1, it can be inferred that

(15) $$\tan\omega \approx \frac{\Delta r}{f}\frac{\sin\left(a\right)}{\cos\left(\beta \right)}$$

When *β* = 0°, the perpendicularity error is minimized. At this point, the impact of r on the perpendicularity error is relatively small and can be ignored.

According to Equations (13) and (15), assuming Δr=0 or Δθ=0°, a is a fixed value, and the angle between the optical axes of the binocular cameras is φ, then φ=90°+β. tanω changes with φ, which can be expressed as

(16) $$\tan\omega =\frac{\tan\omega_{0}}{\cos\left(\varphi -90{}^{\circ}\right)}$$

In the formula, $\omega_{0}$ represents the perpendicularity error of the central axis of a single pile when the camera’s optical axis angle is 90°.

(17) $$\omega =\arctan\left(\frac{\tan\omega_{0}}{\cos\left(\varphi -90{}^{\circ}\right)}\right)$$

**Figure 10:**
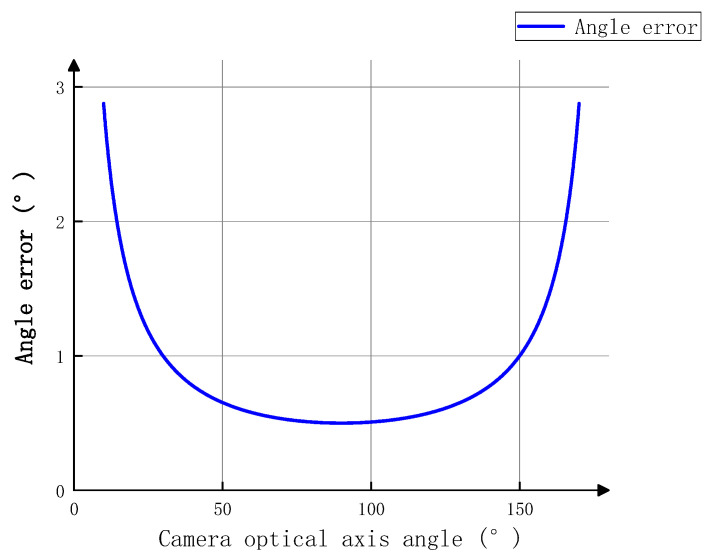
Verticality Error Curve.

In the formula, ω is proportional to $\omega_{0}$, and ω is minimized when φ=90°. When $\omega_{0}$ is taken as the maximum verticality error of 0.5° in the system indicators, the curve of the verticality error ω of a single pile as a function of the camera angle φ is shown in Figure 10.

**Figure 11:**
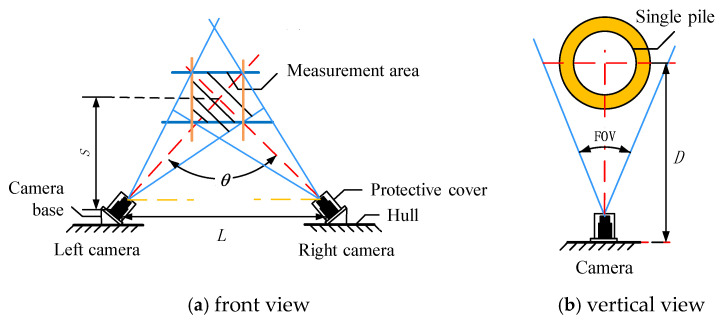
Camera layout for single pile verticality measurement system.

As shown in Figure 10, the angle difference between the perpendicularity error ω and $\omega_{0}$ of the binocular camera within the range of 60°~120° is relatively small, with a maximum difference of 0.08°, which can be ignored. When the distance *S* between the ship platform and the single pile is fixed, the larger the angle θ between the optical axes of the binocular cameras, the greater the distance *L* between the cameras, as shown in Figure 11.

Due to the limited length of the ship’s load-bearing platform, the distance *L* between the cameras cannot be too large. Therefore, in order to ensure that the measurement error of the system’s verticality is small and the distance between the cameras cannot be too large, this article maintains the optical axis angle of the binocular camera within the range of 60°~90°.

When the distance *S* between a single pile and the ship platform is determined, according to the geometric relationship, the distance *L* between the cameras is

(18) $$L=2S\tan\left(\frac{\theta }{2}\right)$$

According to the system measurement indicators, the range of distance *S* between a single pile and the ship platform is 30 m~50 m. When the range of the binocular camera optical axis angle x is 60°~90°, the range of camera spacing *L* that meets the conditions is 58 m~60 m.

In summary, when the optical axes of binocular cameras satisfy the orthogonality condition, the measurement error is minimized. Therefore, under the condition of meeting the technical specifications of the measurement system, this article takes the camera spacing *L* as 60 m, the camera’s optical axis angle θ as 90°, and the distance *S* between the hull and the single pile as 30 m as the research objective. According to the Pythagorean theorem, the distance *D* between a single camera and a single pile is

(19) $$D=\sqrt{S^{2}+{\left(\frac{L}{2}\right)}^{2}}=\sqrt{30^{2}+{\left(\frac{60}{2}\right)}^{2}}=42\;m$$

## 4. Simulated Test

**Figure 12:**
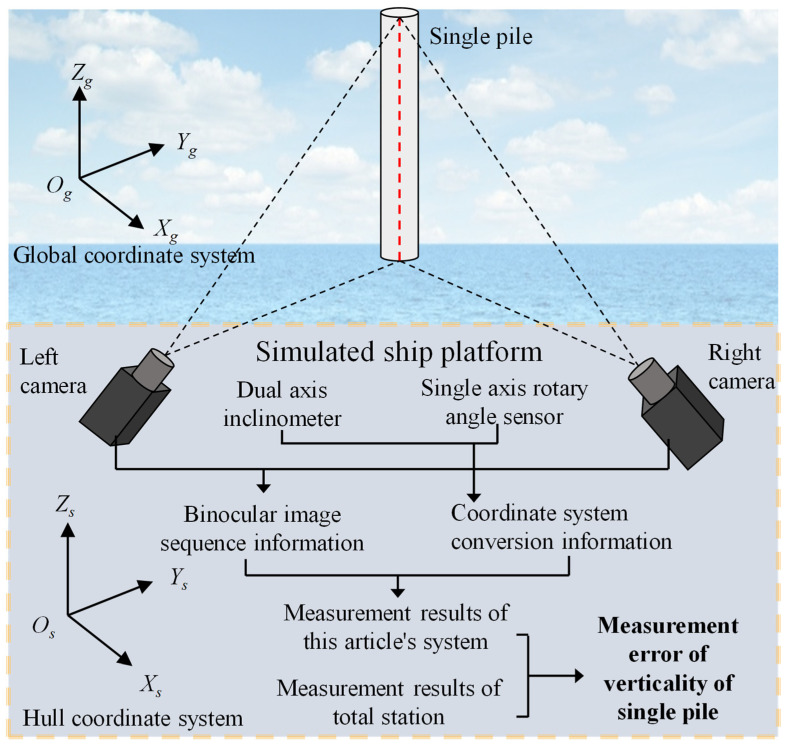
Overall structural layout of the testing experiment.

The overall structural layout of the testing experiment is shown in Figure 12.

The dual axis inclinometer provides angle information for two axes, while the single axis inclinometer provides angle data for the third axis. When the inclinometer and protractor are fixed together with the binocular camera and the simulated ship platform, these devices form a rigid structure. The angle data output from inclinometers and angle sensors can be regarded as providing complete three-axis attitude data, which provides data support for obtaining the rotational mapping relationship between the simulated ship platform and the global coordinate system.

**Figure 13:**
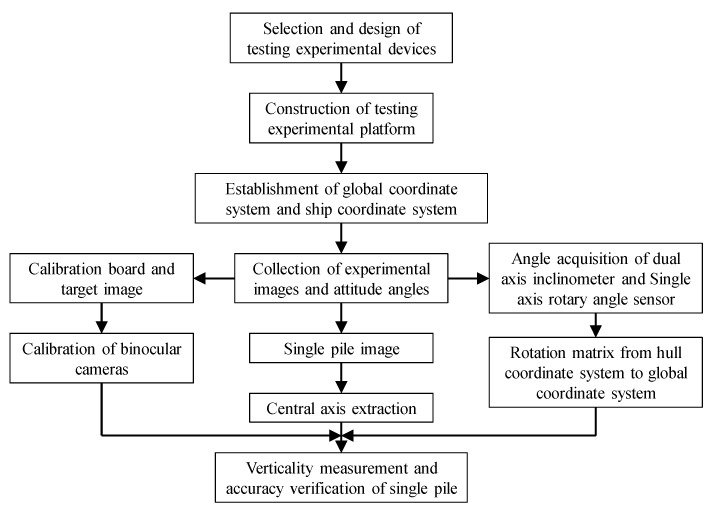
Test experiment process.

In order to meet the requirements of the testing experiment, the testing experiment process designed in this article is shown in Figure 13.

**Figure 14:**
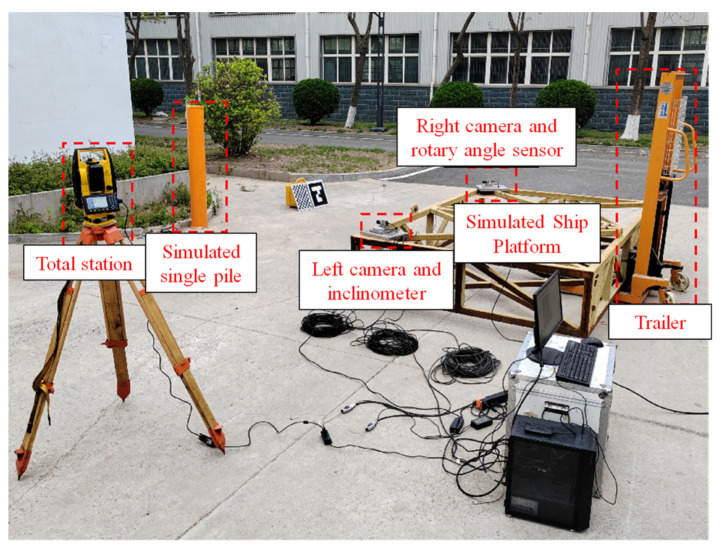
Physical image of experimental platform.

Based on the technical specifications of the measurement system and the camera layout parameters determined in Section 3.5, an experimental platform was built with an equal scale reduction of 20:1, as shown in Figure 14. The platform mainly consists of two high-resolution industrial cameras, two 25 mm lenses, one dual axis inclinometer, one single axis angle meter, one total station, one simulated single pile, one simulated ship platform, one trailer, one GP400 checkerboard calibration board, and several ArUco code targets.

In order to better fit the actual measurement scenario, this article constrained the experimental parameters in the measurement system during the experimental process.

(a) Simulate a single pile

During the process of single-pile driving at sea, the pile body is fixed by a positioning platform, resulting in irrelevant interference information appearing at the bottom of the camera image, and ultimately only a portion of the pile body remains on the sea surface. Therefore, this article selects a PVC pipe with a height of 1.5 m and a diameter of 20 cm as a simulated single pile and simulates the visual interference effect of the positioning platform with a dark background at the bottom of the pile. Due to the constraint of camera field of view, the actual camera imaging shows more of the single color part of the pile, and the simulated single pile surface is designed in a uniform engineering orange color.

(b) The distance between binocular cameras and the angle between the camera’s optical axis

Due to the limitations of simulating ship structures, the distance between the two cameras is approximately 3 m. The cameras are fixed to the simulated ship and placed on the side of the simulated ship near the single pile. The angle between the optical axes of the two cameras is adjusted to be close to orthogonal, and the simulated single pile is ensured to be located at the intersection center of the binocular camera field of view.

(c) Simulate the dynamic process of ship hull

Using trailer control to simulate the lifting and movement of a ship’s body, in order to simulate the shaking of a ship’s body in the ocean. Due to the uncertain effects of sea breeze and waves on the hull, this article controls the lifting frequency and angle of the trailer within a rough range to simulate the actual dynamic process of the hull at sea as much as possible.

During the experiment, a static simulated single pile was selected as the experimental object, and the prism-free mode of the total station was used to laser dot the single pile, obtaining three-dimensional coordinate data of eight points on the same horizontal plane of the single pile. Using the principle of least squares, the cross-section of the single pile was fitted to obtain the three-dimensional coordinates of the fitted center [18], and the three-dimensional coordinates of the centers of the top and bottom cross-sections were then determined to obtain the direction vector of the pile’s axis. The verticality information of the single pile in the global coordinate system is calculated as the standard truth value for the experiment and used to verify the accuracy of the measurement system.

### 4.1. Calibration Experiment of Internal Parameters of Binocular Camera

In the internal parameter calibration process of the camera, this article used a chessboard calibration board with a grid edge length of 30 mm and an internal angle point array of 11 × 8. By adjusting the distance and angle between the calibration board and the camera, 30 calibration images were collected by the left and right cameras, respectively, achieving the calibration of internal parameters of the camera. The specific process is as follows:

(1) Thirty images of the calibration board captured by the camera.
(2) Setting the array of interior corner points and the side length dimensions of the chessboard.
(3) Obtaining pixel coordinates of interior corners on a chessboard using corner detection algorithm.
(4) Solving the internal parameters of a camera without distortion.
(5) Using maximum likelihood estimation to improve calibration accuracy.
(6) Using the least squares method to solve the distortion coefficient.
(7) Using maximum likelihood method for overall parameter optimization.
(8) Solving the reprojection error.

**Figure 15:**
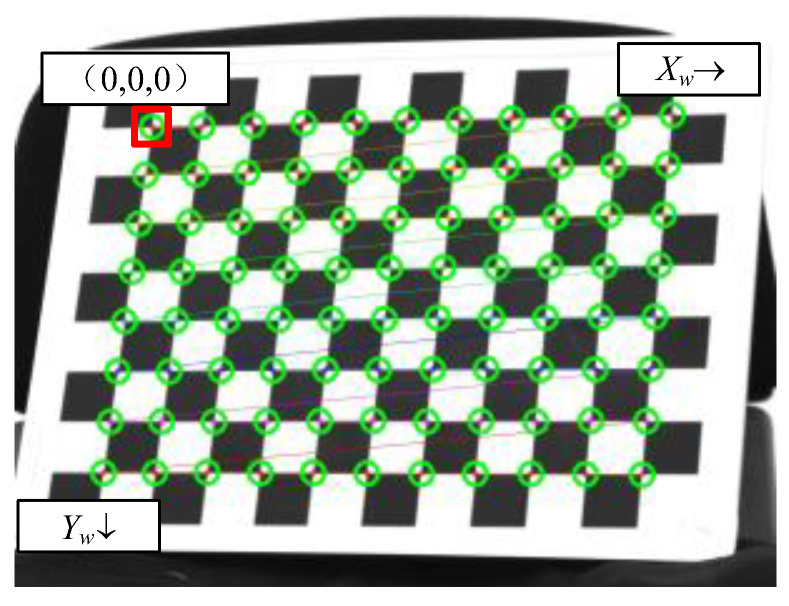
Schematic diagram of chessboard corner detection(The green circle represents the recognized corner position).

After detecting the corner points of the chessboard calibration board, the origin of the world coordinate system is set at the first corner point in the upper left corner of the chessboard. The $Z_{W}$ component of the plane on which the chessboard is located in the world coordinate system $O_{W}\text{-}X_{W}Y_{W}Z_{W}$ is always 0. The plane on which the chessboard is located moves to the right and downwards along the chessboard pattern. The world coordinates of the corner points from the upper left corner to the right and from top to bottom are (0,0,0), (30,0,0), (60,0,0), …, (300,210,0), in millimeters. The schematic diagram of chessboard corner point detection is shown in Figure 15.

By utilizing the mapping relationship between the corner points of the calibration board in the world coordinate system and pixel coordinate system, the internal parameters of the left and right cameras were calibrated. The calibration results are shown in Table 1.

The reprojection error can objectively evaluate the calibration accuracy of the system, and the reprojection error of the binocular camera is shown in Table 2.

According to Table 2, the reprojection errors of the system camera calibration are all less than 0.05 pixels, indicating that the internal parameter calibration of the camera is relatively accurate.

### 4.2. Calibration Experiment of External Parameters of Binocular Camera

Using a total station to establish a global coordinate system on a standard horizontal plane, the simulated ship body is kept in a stationary state. Assuming that the ship body coordinate system is consistent with the global coordinate system, the pose of the binocular camera and the total station are kept relatively stationary to complete the calibration of the external parameters of the binocular camera.

This article uses ArUco code targets with a side length of 30 cm and IDs of 0, 14, 144, and 197 on the central chessboard grid corner points for external parameter calibration of binocular cameras. The specific calibration process is as follows: four ArUco code targets with different IDs are placed in the common view of the left camera and the total station. The total station collects the three-dimensional information of the center point of the ArUco code target with a specific ID, and uses the camera to extract the corresponding pixel information of the target center point. By collecting the three-dimensional and pixel information of four different target center points, the external parameters of the left camera are calibrated. The calibration process for external parameters of the right camera is the same as that of the left camera.

**Figure 16:**
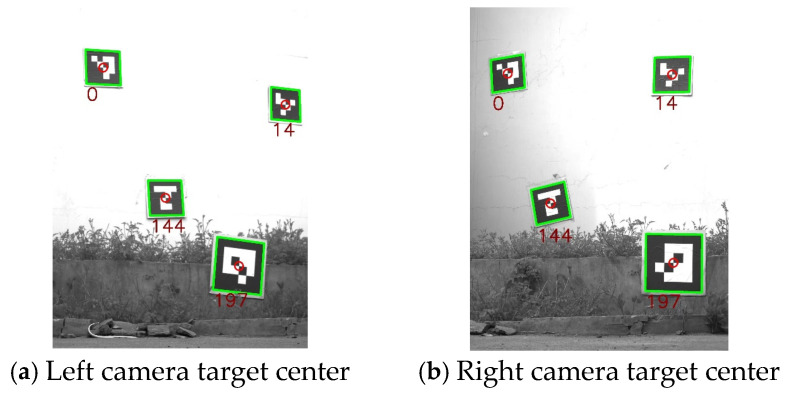
ArUco code target center point (The green box represents the recognized target, and the red circle represents the corner points in the recognized target).

The center points of the four sets of ArUco code targets extracted by the left and right cameras are shown in Figure 16. The three-dimensional point coordinates and pixel information of the target center point extracted by the left and right cameras are shown in Table 3 and Table 4, respectively.

The EPnP algorithm was used to process the data in Table 3 and Table 4, in order to determine the pose mapping matrices from the hull coordinate system to the left and right camera coordinate systems, as shown in Table 5.

### 4.3. Static Simulation Experiment for Measuring the Verticality of a Single Pile

In static simulation experiments, the simulated ship is in a stationary state, and the hull coordinate system is always consistent with the global coordinate system.

Use a total station to obtain the three-dimensional coordinates $P_{1}=\left(4185\;\mathrm{mm},\;5456\;\mathrm{mm},\;24\;\mathrm{mm}\right)$ and $P_{2}=\left(4194\;\mathrm{mm},\;5475\;\mathrm{mm},\;1393\;\mathrm{mm}\right)$ of two points on the central axis of a single pile in the global coordinate system, and then obtain the three-dimensional vector information ${\vec{l}}_{g}^{\prime }=(9,\;19,\;1369)$ of the single pile in the global coordinate system. Calculate the true values of the verticality $\theta_{\mathrm{Vertcality}}^{\prime }$, roll angle $\theta_{\mathrm{Roll}}^{\prime }$, and pitch angle $\theta_{\mathrm{Pitch}}^{\prime }$ of the single pile.

(20) $$\left\{\begin{array}{c} \theta_{\mathrm{Vertcality}}^{\prime }=\arctan\left(\frac{\left|1369\right|}{\sqrt{9^{2}+19^{2}}}\right)=89.1{}^{\circ}\\ \theta_{\mathrm{Roll}}^{\prime }=\arctan\left(\frac{1369}{9}\right)=89.6{}^{\circ}\\ \theta_{\mathrm{Pitch}}^{\prime }=\arctan\left(\frac{1369}{19}\right)=89.2{}^{\circ} \end{array}\right.$$

Using measurement software to extract the central axis of a single pile from the left and right camera images, calculate the vector information $\vec{l_{g}}=\left(7.23\times 10^{12},\;2.76\times 10^{13},\;1.61\times 10^{15}\right)$ of the single pile in the global coordinate system, and calculate the verticality $\theta_{\mathrm{Vertcality}}$, roll angle $\theta_{\mathrm{Roll}}$, and pitch angle $\theta_{\mathrm{Pitch}}$ of the single pile measured by the system.

(21) $$\left\{\begin{array}{c} \theta_{\mathrm{Vertcality}}=\arctan\left(\frac{\left|1.61\times 10^{15}\right|}{\sqrt{{\left(7.23\times 10^{12}\right)}^{2}+{\left(2.76\times 10^{13}\right)}^{2}}}\right)=89.0{}^{\circ}\\ \theta_{\mathrm{Roll}}=\arctan\left(\frac{1.61\times 10^{15}}{7.23\times 10^{12}}\right)=89.7{}^{\circ}\\ \theta_{\mathrm{Pitch}}=\arctan\left(\frac{1.61\times 10^{15}}{2.76\times 10^{13}}\right)=89.0{}^{\circ} \end{array}\right.$$

According to the comparative analysis of the data from Equations (21) and (20), it can be concluded that the verticality error of the system is −0.1°, and the roll angle and pitch angle errors are 0.1° and −0.2°, respectively.

Change the posture of the simulated single pile and repeat the above process five times. The recorded verticality measurement results and errors of the single pile measured by the system as shown in Table 6, and the roll angle and pitch angle measurement results and errors as shown in Table 7.

According to the measurement results and errors of static simulation experiments, the maximum absolute error of the verticality of a single pile is 0.2°, the maximum absolute error of the roll angle is 0.3°, and the maximum absolute error of the pitch angle is 0.3°. The verticality measurement accuracy meets the requirements of the measurement technical indicators.

### 4.4. Dynamic Simulation Experiment for Verticality Measurement of Single Pile

This article combines the angle data provided by a dual-axis inclinometer and a single-axis inclinometer to calculate the rotation matrix from the hull coordinate system to the global coordinate system, in order to complete the experiment of simulating the dynamic process of the ship and measuring the verticality of a single pile.

#### 4.4.1. Acquisition of Hull Coordinate System to Global Coordinate System

Simulate the initial state of the ship in a stationary state, establish a global coordinate system using a total station, and record the three-axis attitude angle output $\theta_{0}=\left(a_{0},\beta_{0},\kappa_{0}\right)$ when the hull coordinate system is the same as the global coordinate system. Change the posture of the simulated ship, use a binocular camera to capture the sequence of single-pile images at this time, and record the three-axis posture angle output $\theta_{i}=\left(a_{i},\beta_{i},\kappa_{i}\right)$, where i=1,2,… represents the *i*-th change in the posture of the simulated ship.

When the three-axis attitude angle outputs are all 0°, the attitude coordinate system is the reference coordinate system $O_{b}\text{-}X_{b}Y_{b}Z_{b}$. When the angle output of the three-axis attitude data is not 0°, according to Equation (6), the rotation matrix $R_{z_{i}b},i=0,1,2,\ldots$ from the attitude coordinate system to the reference coordinate system can be obtained.

(22) $$\left\{\begin{array}{c} \omega_{i}=-\arcsin\left(\frac{\sin\beta_{i}}{\cos\alpha_{i}}\right)\\ \phi_{i}=\alpha_{i} \end{array}\right.$$

(23) $$R_{z_{i}b}=\left[\begin{array}{ccc} \cos\kappa_{i}\cos\varphi_{i} & \cos\kappa_{i}\sin\varphi_{i}\sin\omega_{i}-\sin\kappa_{i}\cos\omega_{i} & \sin\kappa_{i}\sin\omega_{i}+\cos\kappa_{i}\sin\varphi_{i}\cos\omega_{i}\\ \sin\kappa_{i}\cos\varphi_{i} & \cos\kappa_{i}\cos\omega_{i}+\sin\kappa_{i}\sin\varphi_{i}\sin\omega_{i} & \sin\kappa_{i}\sin\varphi_{i}\cos\omega_{i}-\cos\kappa_{i}\sin\omega_{i}\\ -\sin\varphi_{i} & \cos\varphi_{i}\sin\omega_{i} & \cos\varphi_{i}\cos\omega_{i} \end{array}\right]$$

**Figure 17:**
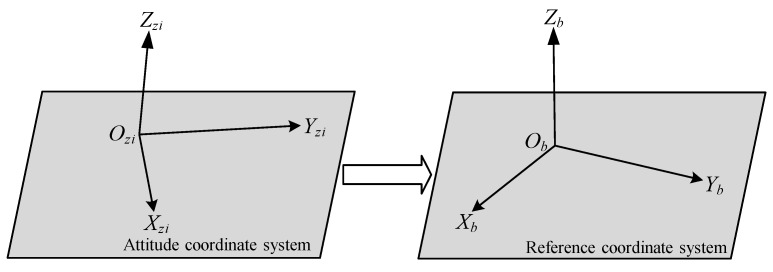
Diagram of conversion from attitude coordinate system to reference coordinate system.

The schematic diagram of the transformation from the attitude coordinate system to the reference coordinate system is shown in Figure 17.

Without considering the translation matrix, according to the rigid body transformation, it can be concluded that

(24) $$\left[\begin{array}{c} X_{b}\\ Y_{b}\\ Z_{b} \end{array}\right]=R_{z_{i}b}\left[\begin{array}{c} X_{z_{i}}\\ Y_{z_{i}}\\ Z_{z_{i}} \end{array}\right],i=0,1,2,\ldots$$

According to Equation (24), after the *i*-th change in the ship’s attitude, the transformation relationship from the attitude coordinate system $O_{zi}\text{-}X_{zi}Y_{zi}Z_{zi}$ to the attitude coordinate system $O_{z0}\text{-}X_{z0}Y_{z0}Z_{z0}$ in the simulated ship’s initial stationary state is as follows:

(25) $$\left[\begin{array}{c} X_{z_{0}}\\ Y_{z_{0}}\\ Z_{z_{0}} \end{array}\right]=R_{z_{0}b}^{-1}R_{z_{i}b}\left[\begin{array}{c} X_{z_{i}}\\ Y_{z_{i}}\\ Z_{z_{i}} \end{array}\right],i=1,2,\ldots$$

Due to the fact that the dual-axis inclinometer, single-axis inclinometer, and the hull form a rigid body, the attitude coordinate system and the hull coordinate system are always in a relatively static state, and the reference coordinate system and the global coordinate system remain unchanged. Therefore, the rotation matrix $R_{sg}=R_{z_{0}b}^{-1}R_{z_{i}b}$ from the hull coordinate system to the global coordinate system can be obtained.

#### 4.4.2. Dynamic Simulation Experiment on Verticality Measurement of Single Pile

Place the single pile in a stationary position and use a total station to record the true verticality value $\theta_{\mathrm{Verticality}}^{\prime }$ of the single pile as 83.3°, the true roll angle $\theta_{\mathrm{Roll}}^{\prime }$ and pitch angle $\theta_{\mathrm{Pitch}}^{\prime }$ as −84.1° and 86.8°, respectively. Record the initial three-axis angle output $\theta_{0}=\left(0.69{}^{\circ},\;-0.82{}^{\circ},\;84.31{}^{\circ}\right)$ of the static simulation ship platform at this time. The rotation relationship between the attitude coordinate system $O_{z0}\text{-}X_{z0}Y_{z0}Z_{z0}$ and the reference coordinate system $O_{b}\text{-}X_{b}Y_{b}Z_{b}$ is known as

(26) $$R_{z_{0}b}=\left[\begin{array}{ccc} 0.0991 & 0.9950 & 0.0130\\ -0.9950 & 0.0989 & 0.0134\\ 0.0120 & -0.0143 & 0.9998 \end{array}\right]$$

By changing the posture of the simulated ship and using a binocular camera to capture a sequence of single-pile images, the three-dimensional vector information of the single pile in the hull coordinate system can be calculated as $\vec{l_{s}}=\left(-1.61\times 10^{14},\;5.53\times 10^{12},\;9.57\times 10^{14}\right)$. At the same time, the three-axis angle output $\theta_{1}=\left(-2.95{}^{\circ},\;-3.04{}^{\circ},\;88.31{}^{\circ}\right)$ is recorded. It can be seen that the rotation matrix from the attitude sensor coordinate system $O_{zi}\text{-}X_{zi}Y_{zi}Z_{zi}$ to the reference coordinate system $O_{b}\text{-}X_{b}Y_{b}Z_{b}$ is

(27) $$R_{z_{1}b}=\left[\begin{array}{ccc} 0.0294 & 0.9981 & 0.0545\\ -0.9982 & 0.0322 & -0.0498\\ -0.0515 & -0.0530 & 0.9973 \end{array}\right]$$

Based on Equations (26) and (27), it can be concluded that the rotation matrix $R_{sg}$ from the hull coordinate system to the global coordinate system is

(28) $$R_{sg}=R_{z_{0}b}^{-1}R_{z_{1}b}=\left[\begin{array}{ccc} -0.9956 & 0.0663 & -0.0670\\ -0.0687 & -0.9970 & -0.0351\\ -0.0644 & -0.0395 & 0.9971 \end{array}\right]$$

The three-dimensional vector $\vec{l_{g}}$ measured in the hull coordinate system is converted to the global coordinate system as follows:

(29) $${\left(\vec{l_{g}}\right)}^{T}=R_{sg}{\left(\vec{l_{s}}\right)}^{T}=\left[\begin{array}{ccc} -0.9956 & 0.0663 & -0.0670\\ -0.0687 & -0.9970 & -0.0351\\ -0.0644 & -0.0395 & 0.9971 \end{array}\right]\left[\begin{array}{c} -1.61\times 10^{14}\\ 5.53\times 10^{12}\\ 9.57\times 10^{14} \end{array}\right]$$

If the three-dimensional vector $\vec{l_{g}}=\left(-9.57\times 10^{13},5.01\times 10^{13},9.64\times 10^{14}\right)$ of a single pile in the global coordinate system is known, the verticality $\theta_{\mathrm{Verticality}}$ of the single pile measured by the system is 83.6°, and the roll angle $\theta_{\mathrm{Roll}}$ and pitch angle $\theta_{\mathrm{Pitch}}$ are −84.3° and 87.0°, respectively. When comparing it with the true value of the single-pile data measured by the total station, it can be seen that the verticality error of the system is 0.3°, and the measurement errors of roll angle and pitch angle are −0.2° and 0.2°.

According to the above process, the verticality of simulated single piles is measured in two different postures: one in which the single pile is placed almost vertically, and another in which it is placed in an arbitrary posture. Five repeated measurements were taken for the verticality of the single pile in each posture. The measurement results and errors of the verticality, roll angle, and pitch angle of the single pile measured by the system in these two postures are shown in Table 8 and Table 9.

According to the measurement results and errors of dynamic simulation experiments, the maximum absolute error of the verticality of a single pile is 0.4°, the maximum absolute error of the roll angle is 0.3°, and the maximum absolute error of the pitch angle is 0.3°.

## 5. Conclusions and Prospects

### 5.1. Conclusions

In the field of offshore wind power, single piles provide stable support for wind turbines, ensuring their safe and efficient operation in harsh marine environments. With the rapid development of the wind power industry, stricter requirements have been put forward for the accuracy and efficiency of verticality measurement of single piles. This article studies the online measurement method of verticality of offshore wind power single piles based on binocular vision technology, and establishes a mathematical model and error analysis model for measuring the verticality of single piles. We have built a small-scale proportional simulation experimental platform and completed the design of the experimental software. By conducting static and dynamic simulation experiments on the hull, the system measurement results were compared with the total station measurement results. In the static simulation experiment, the maximum absolute error of the verticality of a single pile was 0.2°, the maximum absolute error of the roll angle was 0.3°, and the maximum absolute error of the pitch angle was 0.3°; In the dynamic simulation experiment, the maximum absolute error of the verticality of a single pile is 0.4°, the maximum absolute error of the roll angle is 0.3°, and the maximum absolute error of the pitch angle is 0.3°. This verifies that the verticality measurement accuracy of the measurement system meets the requirements of the system indicators and is expected to provide real-time data for single-pile attitude adjustment.

### 5.2. Prospects

This article establishes a mathematical model and error analysis model for measuring the verticality of offshore wind power single piles based on principles of mathematical physics. However, due to limitations in the current project research progress and measurement conditions, experiments have not been conducted in real marine environments. Considering that factors such as sea visibility, water surface reflection, and environmental interference only affect the accuracy of image measurement algorithms, and the fact that the model in this paper is derived based on mathematical and physical principles, the correctness of the model was verified through simulation experiments. The validation of image measurement algorithms will be carried out in real marine environments, which is beyond the scope of this article.

## Figures and Tables

**Table 1:** Internal parameters and distortion coefficients of binocular cameras.

Camera Parameters	Left Camera	Right Camera
Internal parameter matrix	$\left[\begin{array}{ccc} 7360.27 & 0 & 1043.91\\ 0 & 7359.57 & 2071.70\\ 0 & 0 & 1 \end{array}\right]$	$\left[\begin{array}{ccc} 7314.79 & 0 & 1068.55\\ 0 & 7312.89 & 2069.58\\ 0 & 0 & 1 \end{array}\right]$
Radial distortion coefficient	$\left[\begin{array}{ccc} -0.0992 & 0.6598 & -0.0215 \end{array}\right]$	$\left[\begin{array}{ccc} -0.0784 & 0.5219 & -0.0397 \end{array}\right]$
Tangential distortion coefficient	$\left[\begin{array}{cc} 0.0003\; & -0.0001 \end{array}\right]$	$\left[\begin{array}{cc} 0.0002 & 0.0003 \end{array}\right]$

**Table 2:** Calculation results of reprojection error.

	Left Camera (Pixel)	Right Camera (Pixel)
Reprojection error	0.031	0.048

**Table 3:** Three-dimensional coordinates and pixel information of the center point of the left camera target.

ArUco’s ID	Center Point Coordinates (mm)	Pixel Information
0	(8971,6819,2460)	(427.16,1473.50)
14	(7966,8547,2242)	(1973.33,1788.52)
144	(8644,7377,1190)	(955.39,2580.13)
197	(6630,5617,370)	(1451.83,3166.67)

**Table 4:** Three-dimensional coordinates and pixel information of the center point of the right camera target.

ArUco’s ID	Center Point Coordinates (mm)	Pixel Information
0	(10124,4835,2449)	(315.03,1584.92)
14	(9279,6291,2296)	(1689.68,1593.21)
144	(9877,5232,1112)	(672.09,2673.25)
197	(6766,5373,355)	(1710.23,3172.72)

**Table 5:** Rotation and translation matrices from the hull coordinate system to the left and right camera coordinate systems.

Coordinate System Transformation	Rotation Matrix	Translation Matrix (mm)
Hull coordinate system to left camera coordinate system	$R_{h\_cl}=\left[\begin{array}{ccc} 0.6396 & -0.7686 & -0.0055\\ 0.1524 & 0.1338 & -0.9792\\ 0.7534 & 0.6255 & 0.2028 \end{array}\right]$	$T_{h\_cl}=\left[\begin{array}{c} 1829\\ -527\\ -2692 \end{array}\right]$
Hull coordinate system to right camera coordinate system	$R_{h\_cr}=\left[\begin{array}{ccc} 0.9687 & -0.2479 & -0.0096\\ 0.0427 & 0.2046 & -0.9779\\ 0.2445 & 0.9469 & 0.2088 \end{array}\right]$	$T_{h\_cr}=\left[\begin{array}{c} -3063\\ -464\\ -2478 \end{array}\right]$

**Table 6:** Verticality measurement results and errors. Unit: °.

Serial Number	Verticality Calculation	True Verticality Value	Verticality Error
1	86.8	86.6	0.2
2	84.0	84.1	−0.1
3	86.6	86.5	0.1
4	87.8	87.9	−0.1
5	88.4	88.2	0.2

**Table 7:** Measurement results and errors of roll angle and pitch angle. Unit: °.

Serial Number	Angle Calculation Value		True Angle Value		Angle Error Value	
	Roll Angle	Pitch Angle	Roll Angle	Pitch Angle	Roll Angle	Pitch Angle
1	88.2	87.3	88.0	87.2	0.2	0.1
2	84.5	−87.6	84.7	−87.5	−0.2	−0.1
3	−86.9	−88.6	−86.9	−88.3	0.0	−0.3
4	−88.3	−88.6	−88.6	−85.5	0.3	−0.1
5	88.4	−89.9	88.2	−89.9	0.2	0.0

**Table 8:** The measurement results and errors of verticality, roll angle, and pitch angle with true values of 88.5°, −89.2°, and −88.7° Unit: °.

Serial Number	Calculated Angle			Angular Error		
	Verticality	Roll Angle	Pitch Angle	Verticality	Roll Angle	Pitch Angle
1	88.4	−89.0	−88.7	−0.1	0.2	0.0
2	88.5	−89.4	−88.6	0.0	−0.2	0.1
3	88.2	−89.3	−88.4	−0.3	0.1	0.3
4	88.4	−89.5	−88.5	−0.1	−0.3	0.2
5	88.7	−89.4	−88.9	0.2	−0.2	−0.2

**Table 9:** Measurement results and errors for verticality, roll angle, and pitch angle with true values of 82.6°, 83.0°, and 87.6°. Unit: °.

Serial Number	Calculated Angle			Angular Error		
	Verticality	Roll Angle	Pitch Angle	Verticality	Roll Angle	Pitch Angle
1	82.5	82.9	87.5	−0.1	−0.1	−0.1
2	82.9	83.2	87.7	0.2	0.2	0.1
3	83.0	83.3	87.8	0.4	0.3	0.2
4	82.2	82.7	87.3	−0.4	−0.3	−0.3
5	82.4	82.8	87.7	−0.2	−0.2	0.1

## Data Availability

The data used to support the finding of this study are available from the corresponding author upon request.
